# Immunohistochemical expression of HMGB1 and related proteins in the skin as a possible tool for determining post-mortem interval: a preclinical study

**DOI:** 10.1007/s12024-023-00634-1

**Published:** 2023-07-25

**Authors:** Fabio De-Giorgio, Eva Bergamin, Alfonso Baldi, Roberto Gatta, Vincenzo L. Pascali

**Affiliations:** 1 Fondazione Policlinico Universitario A. Gemelli IRCCS, Rome, Italy; 2 Department of Healthcare Surveillance and Bioethics, Section of Legal Medicine, Università Cattolica del Sacro Cuore, Rome, Italy; 3 Department of Environmental, Biological and Pharmaceutical Sciences and Technologies, Università degli Studi della Campania Luigi Vanvitelli, Caserta, Italy; 4 Department of Oncology, Lausanne University Hospital, Lausanne, Switzerland

**Keywords:** Immunohistochemistry, Histology, HMGB1, Post-mortem interval, Post-mortem investigation, Autopsy, Forensics

## Abstract

Determining the post-mortem interval (PMI) is one of forensic pathology’s primary objectives and one of its most challenging tasks. Numerous studies have demonstrated the accuracy of histomorphology and immunohistochemical investigations in determining the time of death. Nevertheless, the skin, a robust and easy-to-remove tissue, has only been partially analyzed so far. By studying 20 adult male mice, we tried to determine whether post-mortem immunohistochemical detection in the skin of HMGB1 proteins and associated components (Beclin1 and RAGE) could be used for this purpose. We discovered that nuclear HMGB1 overexpression indicates that death occurred within the previous 12 h, nuclear HMGB1 negativization with high cytoplasmic HMGB1 intensity indicates that death occurred between 12 and 36 h earlier and cytoplasmic HMGB1 negativization indicates that more than 48 h have passed since death. RAGE and Beclin1 levels in the cytoplasm also decreased with time. The latter proteins’ negativization might indicate that more than 24 and 36 h, respectively, have passed from the time of death. These indicators might potentially be helpful in forensic practice for determining the PMI using immunohistochemistry.

## Introduction

One of the main goals of forensic pathology, as well as one of its most difficult tasks, is the determination of time since death or post-mortem interval (PMI). Undoubtedly, this parameter is of crucial importance, especially in relation to criminal investigations, yet many studies on the topic discuss only the time dependency of post-mortem parameters that have little application to real forensic practice [1–3]. Despite the development in recent years of several new approaches to the assessment of PMI [3, 4], traditional methods, based on the study of *rigor*, *algor* and *livor mortis*, are still the most commonly used. These factors, however, are largely the result of physical and chemical processes that occur in the post-mortem period and may be affected by a wide range of individual and environmental factors (i.e. ambient temperature, age, gender and physiological and pathological states). As a result, traditional approaches are often characterized by inaccuracy, a lack of reliability and consequently limitations in their application [1–3].

The new approaches aiming to develop more precise PMI estimates vary not only in terms of the biological processes considered but also in terms of their scientific rigour and the data validation methods applied. In a recent review, Gelderman et al. [5] examined, in the light of the Daubert criteria, the reliability of several approaches used to estimate PMI. Of all the approaches considered, only Henssge’s nomogram and forensic entomology met the required criteria. The need to overcome the limitations of methodologies currently used for calculating PMI and to reduce the temporal uncertainty, which is generally far too wide, has prompted us to consider immunohistochemistry as a potential approach that may provide more objective data for estimating time of death.

Histomorphological and immunohistochemical analysis of the skin has already shown significant potential in PMI estimation, and multiple studies confirm its reliability [6–22].

The skin is more resilient and robust than other soft tissues, and it may be able to withstand abiotic and transformative processes better, thereby providing significant data for PMI assessment. Furthermore, skin is readily accessible for sampling, and immunohistochemistry investigations are relatively easy to carry out and do not require advanced investigative procedures. Skin tissue analyses have the potential to provide useful information concerning not only PMI but also lesions and pathologies related to cause of death.

Post-mortem transformation typically causes cell necrosis, which results in the release of the chromatin protein high mobility group box-1 (HMGB1) [15, 23, 24].

HMGB1 is found in many eukaryotic cells and has an amino acid sequence that is substantially conserved across species. Its biological roles include that of intracellular transcription regulator, and following necrosis, it translocates outside of the nucleus and is released by macrophages. To date, only a few studies have been conducted with the aim of investigating the role of this biomarker in the context of PMI estimation [23, 24].

The purpose of this study was thus to examine the possible applicability of post-mortem histological alterations in the skin, in conjunction with the immunohistochemical detection of HMGB1 proteins and related factors (Beclin1 and RAGE), for estimating the time elapsed since death, using 20 adult male mice.

## Materials and methods

### Animal specimens

Twenty adult male albino mice were used in this study (age ranged from 8 to 9 months). The mice were classified into two groups (10 mice per group) based on the time and day of post-mortem skin biopsy collection. All 20 animals were dissected to obtain full-thickness skin samples at different intervals (0, 12-, 24-, 36- and 48-h post-mortem). Therefore, 4 animals for each time point were selected.

### Histopathology and immunohistochemical study

The skin tissue samples of 20 mice were obtained at 0, 12-, 24-, 36- and 48-h post-mortem (hpm). Mice were sacrificed by cervical dislocation and all the skin biopsies were taken from the dorsal region of the animals. All samples were stained with hematoxylin and eosin (H&E) and anatomical integrity was verified. Subsequently, immunohistochemical analyses were carried out on serial sections, using the following antibodies:

- Recombinant anti-HMGB1 antibody [EPR3507] (ab79823)
- Recombinant anti-Beclin 1 antibody [EPR20473] (ab210498)
- Recombinant anti-RAGE antibody [EPR21171] (ab216329)

Serial sections of 5–7 μm were obtained from each sample, and the most representative sections were selected after observation with H&E staining. All sections were then washed with xylene and fully rehydrated using a sequence of decreasing alcohols before being washed with phosphate-buffered saline (PBS). PBS was used for all subsequent washes and for antibody dilution. Tissue sections were sequentially treated with 3% hydrogen peroxide in aqueous solution and blocked with 6% milk in PBS. The slides were then incubated for 1 h at room temperature with each of the specified antibodies, at a final dilution of 1:100. Following three PBS washes aimed at removing excess antibodies, the slides were incubated with the UltraTek HRP secondary antibody (ScyTek Laboratories, Logan, UT, USA) for 1 h at room temperature. The ABC technique (Vector Laboratories) was applied to all slides for 30 min at room temperature. Diaminobenzidine (ScyTek Laboratories, Logan, UT, USA) was used as the final chromogen, and hematoxylin was used as a contrast agent. For each tissue section, a negative control was generated without the primary antibody. All samples were processed under the same conditions. The cellular expression levels of HMGB1, Beclin 1 and RAGE per field (10X) were calculated under the microscope, with two different observers comparing samples, and they were characterized as follows: *score 0* (absent), *score 1* (low or moderate) and *score 2* (high). An average of 22 fields was observed for each sample. The temporal evolution of the HMBG1 was statistically tested with the Jonckheere’s trend test. The level of concordance, expressed as the percentage of agreement between the observers, was 95%. For the remaining specimens, the score was obtained after collegial revision and agreement.

## Results

**Fig. 1:**
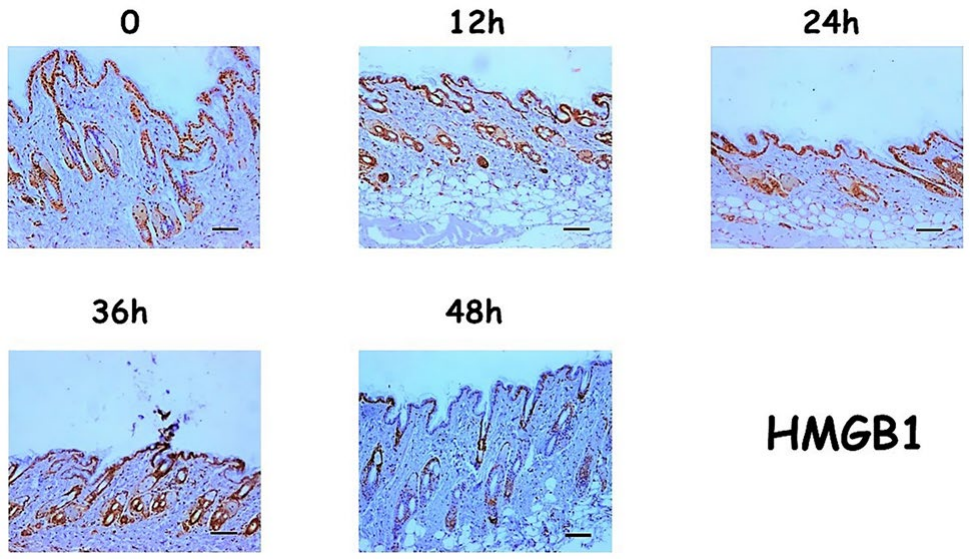
Expression patterns for HMGB1 (Scale bar = 100µ)

We examined the skin samples of 20 adult male mice with the aim of identifying possible patterns in the post-mortem behaviour of specific proteins, particularly HMGB1, RAGE and Beclin-1. Significant quantitative alterations were found in the expression of the proteins studied, as well as a signal translocation for HMGB1 from the nucleus to the cytoplasm. At time 0 hpm, the expression of HMGB1 was found to be high (score 2) and at nuclear localization. Twelve hours after death, HMGB1 expression was no longer at the nuclear level, but rather at the cellular level, still with high intensity. At 24 and 36 hpm, both the type and degree of expression remained unchanged. However, at 48 hpm, the intensity of expression was reduced relative to the previous time periods, although it was still cytoplasmic; this trend was considered significant (*p* < 0.05) at the Jonckheere’s (*p* = 5.995*10^-16). Figure 1 shows an example of the HMGB1 expression in the skin epithelium.

**Fig. 2:**
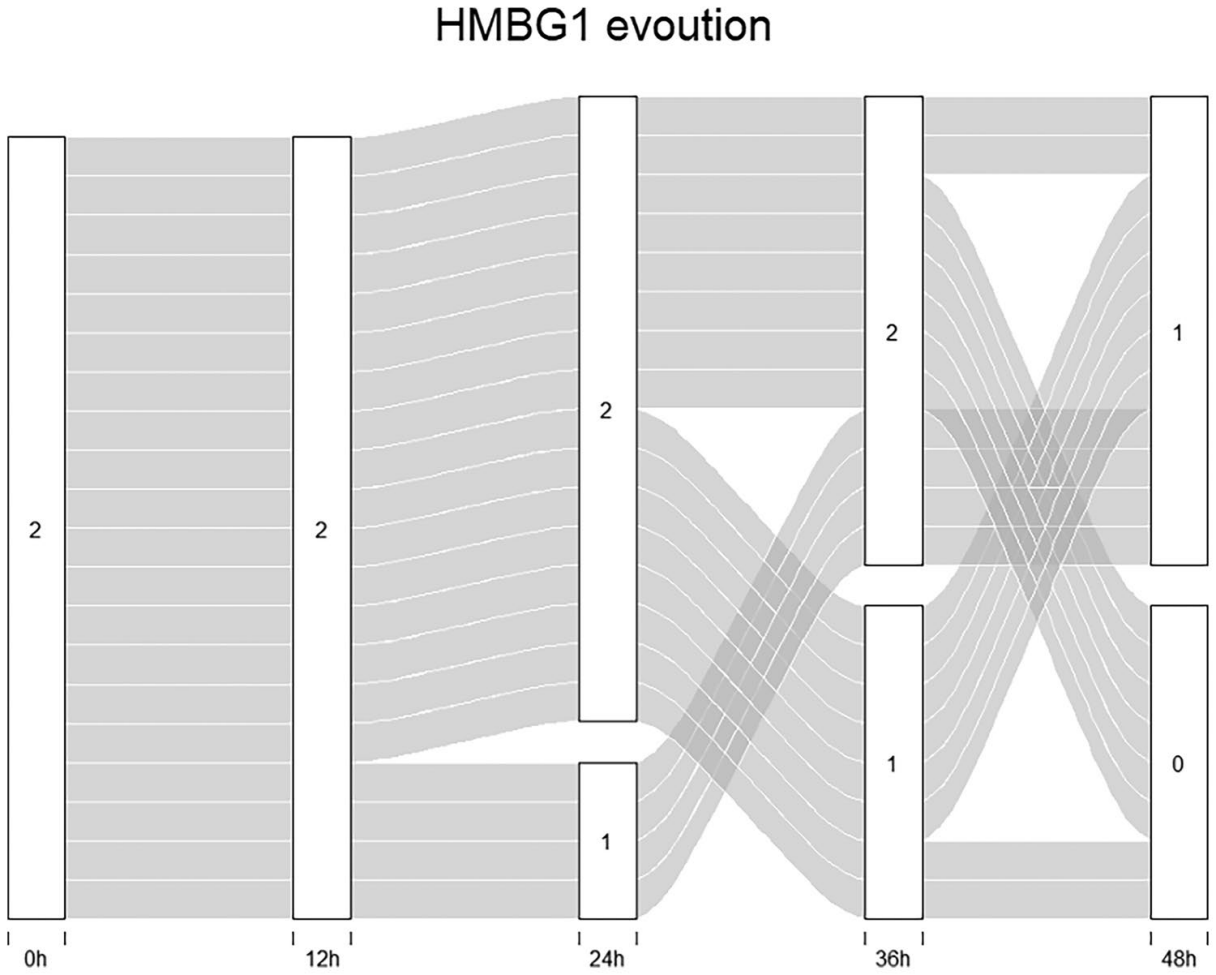
Alluvial graph: the graph shows the temporal evolution of the HMBG1 classes for each subject, at the different post-mortem time points (0, 12, 24, 36, 48 h)

Figure 2 shows the temporal evolution of the HMGB1 classes for each subject.

**Fig. 3:**
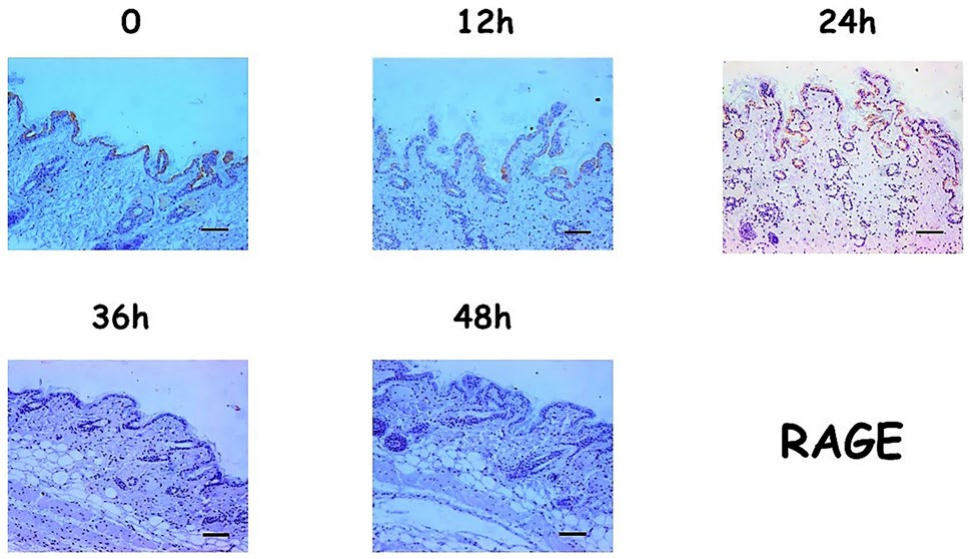
Expression patterns for RAGE (Scale bar = 100µ)

The expression patterns observed for RAGE and Beclin1 were different. At time 0, RAGE expression was high and cytoplasmic in the skin epithelium. At 24 and 36 hpm, the expression was still cytoplasmic, but it was found to be lower than that observed at time 0. Finally, after 48 h, the protein’s expression was completely absent (Fig. 3).

**Fig. 4:**
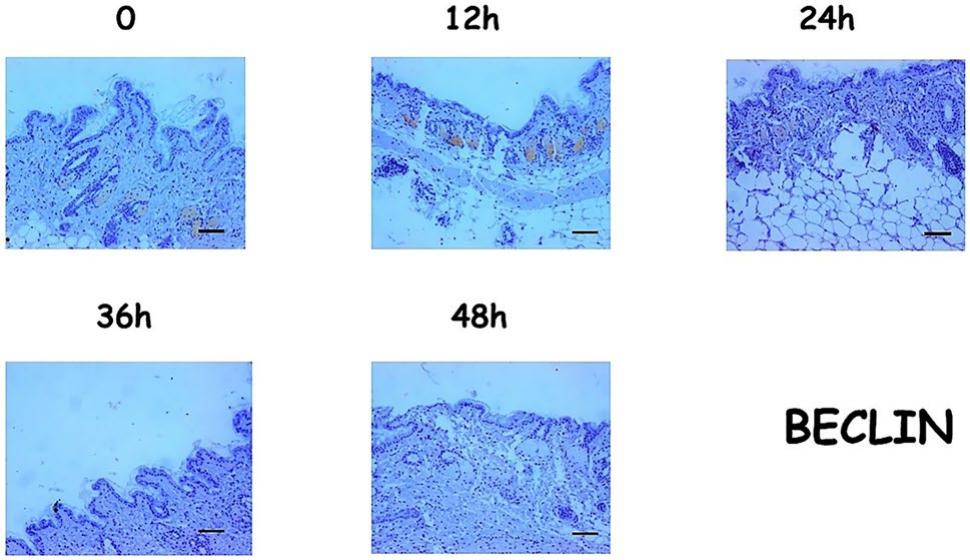
Expression patterns for Beclin1 (Scale bar = 100µ)

In contrast, at time 0, Beclin1 expression was low in skin appendages (particularly at the level of the sebaceous glands) and almost completely absent in the skin epithelium. At 24 and 36 hpm, Beclin1 expression was high in the appendages and low in the epithelium. Finally, after 48 h, the protein was expressed in neither the appendages nor in the skin epithelium (Fig. 4).

**Fig. 5:**
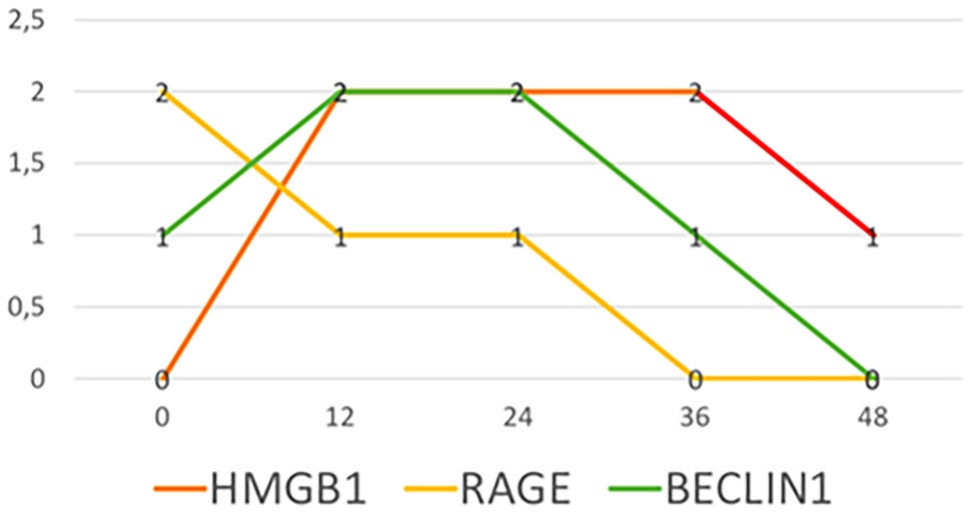
Trends of cytoplasmic expression in mice skin and appendages

Figure 5 shows the trends of cytoplasmic expression in mice skin and appendages for HMGB1, Beclin1 and RAGE. The intensity of HMGB1 at the cytoplasmic level peaked between 12 and 36 hpm, after which, the marker decreased and was no longer visible after 48 h. The other markers, on the other hand, were always present in the cytoplasm, albeit in varying amounts, depending on the different times of the analyses. The negativization of these markers, which occurred at 36 hpm for RAGE and at 48 hpm for Beclin1, is noteworthy (Fig. 5).

All the results of the immunohistochemical examination, including the different expression levels and the sub-cellular localizations of the proteins analysed at the different time points are presented in Table 1.

**Table 1:** Different levels of immunohistochemical expression of the analyzed proteins at the selected time points

**Protein**	**0 hpm**	**12 hpm**	**24 hpm**	**36 hpm**	**48 hpm**
HMGB1	2 (nucleus)	2 (cytoplasm)	2 (cytoplasm)	2 (cytoplasm)	1 (cytoplasm)
RAGE	2 (cytoplasm)	1 (cytoplasm)	1 (cytoplasm)	0	0
BECLIN	1 (annexes)	2 (annexes)	2 (annexes)	1 (annexes)	0

*Hpm* hours post-mortem

## Discussion

The traditional methods of forensic practice are still commonly utilized for estimating PMI. However, these methods are limited in their applicability as the indicators *rigor*, *livor* and *algor mortis* are derived primarily from post-mortem physical and chemical processes and are susceptible to modification by a range of intrinsic and extrinsic factors. This often leads to inaccuracies in interpretation. Any anomalous or unusual course that impacts one or more cadaveric phenomena, which might also mutually impact each other, will have chronological implications and increase the likelihood of inaccuracy. As time passes, the assessment of PMI becomes increasingly approximate.

In recent years, several experimental approaches have been explored to establish a more precise method for assessing PMI. These techniques differ both in nature and in terms of their scientific rigour. A review by Gelderman et al. [5] investigates the reliability of several approaches that are reported in 94 recent Dutch judicial proceedings in which time of death was requested. The methods employed to determine PMI are evaluated in the light of Daubert’s criteria. Only *algor mortis*, as measured by the Henssge nomogram, and forensic entomology provided scientifically sound results that met Daubert’s criteria.

Histopathology has played an important role in the forensic field and is usually primarily used to establish the cause of death [6–30]. Over the years, however, numerous novel approaches have been developed for estimating PMI, with some addressing the domains of histology and immunohistochemistry. Table 2 provides an overview of the literature on the topic. Ideally, a complete evaluation of all measurable changes in the process of human tissue and organ decomposition would reveal the presence of a distinct sequence, which could be used to calculate PMI. However, in practice, this is hard to recognise. Wehner et al. [6–9] investigated whether positive immunoreactions to various antigens (i.e. insulin, glucagon, thyroglobulin and calcitonin) correlated with PMI, assuming that antigen structure changes post-mortem and that the efficacy of protein denaturation staining will decrease with increasing PMI. Among studies that have investigated human tissue and organs, some have focused on the macroscopic and histological changes occurring, post-mortem, in the skin, and these have occasionally been used to determine the time of death under varied settings. For instance, Kovarik et al. [11] investigated the macroscopic and microscopic appearance of the skin in three deceased individuals during the first post-mortem week, under specific climatic conditions. For each case, skin biopsies were obtained from four different sites at 12- to 24-h intervals. The authors observed three major histological changes, including isolated dermal-epidermal separation, eccrine duct necrosis and dermal degeneration, which could potentially be used to estimate PMI in the early post-mortem period. Studies have also shown that skin tissue can provide important information on lesions and diseases, which are frequently associated with both cause of death and PMI. Because the skin is easier to sample and more resilient than other soft tissues, it has the potential to challenge abiotic and transformative factors, thereby providing significant data for measuring PMI. However, only a few studies dealing with the use of skin markers for the determination of PMI have been reported in the literature. Furthermore, as previously mentioned, individual and environmental variables commonly influence PMI and, as a result, restrict the applicability of these approaches. The present study therefore considers animal studies, as animal models are often able to illustrate the basic principles of biological processes. El-Din et al. [15] investigated the possible association between post-mortem skin abnormalities and HMGB1 changes as well as PMI, through the immunohistochemical staining of serum and skin, using both animal (40 adult male albino rats) and human (40 cases) samples. Human forensic autopsies were performed within the first 24 hpm on deceased individuals with a known time of death, whereas mice were dissected at 0, 3, 6, 12 and 24 hpm. The authors identified a specific pattern of HMGB1 expression in mice skin tissue: at 0 hpm, HMGB1 showed a weak positive immunoreaction, a mild positive reaction at 3 and 6 hpm, and moderate and strong positive immunoreactions at 12 and 24 hpm, respectively. These findings revealed a considerable time-dependent increase in serum HMGB1 levels, as well as its overexpression in immunohistochemically stained skin tissue, suggesting that HMGB1 might be a reliable post-mortem marker.

**Table 2:** Post-mortem histomorphological and immunohistochemical investigation as a tool for the PMI estimation: a review of the literature on this topic

**Ref.**	**No. of cases**	**Biological samples**	**Immunohistochemical/histological exams**	**PMI investigated**	**Results**	**Impact on the scientific community**
[22]	147 corpses Studied with external factors (temperature and humidity), environmental factors (closed, open or in water) and factors related to the body (clothes or body) differing in each case	Colloid and follicular cells of the thyroid gland	DAKO EPOS-System: monoclonal mouse antihuman thyroglobulin antibody with diaminobenzidine	From 1 to 21 days after death	Positive immunoreaction for thyroglobulin for up to 5 days, in all cases No reaction after 13 days	Positive correlation between post-mortem changes in the structure of thyroglobulin and the colourability of the same are time-dependent: the denaturation of the protein makes it unsuitable for immunohistochemical staining Immunohistochemical detection of thyroglobulin can be considered a useful method for delimiting the PMI, especially in cases of longer PMI
[23]	128 corpses Studied with external factors (temperature and humidity), environmental factors (closed, open, in water) and factors related to the body (clothes, body) differing in each case	Cells B pancreas	Guinea pig polyclonal antibody (DAKO Company) used for the detection of insulin The biotinylated anti-rabbit-F (ab9) 2 fragment was used as a secondary antibody. Detection was carried out with the avidin–biotin-peroxidase method with 3,39-diaminobenzidine	From 1 to 45 days after death	Positive immunoreaction to insulin for up to 12 days after death; no reaction for longer than 30 days	Positive correlation between post-mortem changes in the structure of insulin and its colourability over time: the denaturation of the protein makes it unsuitable for immunohistochemical staining However, the time limits resulting from the study may change in the event of a significant variation in the environmental conditions related to the discovery of the corpse
[24]	136 corpses Studied with external factors (temperature and humidity), environmental factors (closed, open, in water) and factors related to the body (clothes, body) differing in each case	Thyroid C cells	Immunohistochemical staining with the DAKO-Epos system. Detection of calcitonin with a prediluted rabbit anti-human calcitonin antibody. Detection of antibody binding was carried out with diaminobenzidine	From 1 to 21 days after death	Positive immunoreaction to calcitonin up to 4 days after death. No reaction 13 days after death	Positive correlation between the post-mortem changes in the structure of calcitonin and its colourability over time: the denaturation of the protein makes it unsuitable for immunohistochemical staining However, the time limits resulting from the study may change in the event of a significant variation in the environmental conditions related to the discovery of the corpse
[25]	214 corpses Studied with external factors (temperature and humidity), environmental factors (closed, open, in water) and factors related to the body (clothes, body) differing in each case	Pancreas	Rabbit polyclonal antibody (DAKO Co) for glucagon. Anti-rabbit biotinylated secondary antibody F (ab0) 2. Detection of the avidin–biotin peroxidase complex by method	From 1 to 21 days after death	Positive immunoreaction to glucagon in all cases for up to 6 days. No immunoreactions were found in the cases analysed 14 days after death	Possible use of the immunohistochemical detection of glucagon at different time intervals to estimate PMI
[26]	105 corpses Cases with diabetes mellitus, thyroid disease, or gross pancreatic disease were excluded. External factors varied from case to case	Pancreas and thyroid	Antibodies against insulin, glucagon, thyroglobulin and calcitonin (Kit: universal LSABTM2 Kit/HRP, Rb/Mo, Dako K0675, antibodies: Polyclonal Guinea Pig Anti-Insulin, Dako A0564, Rabbit Monoclonal [EP3070] to Glucagon, Abcam ab92517. Kit: EnVisionTM + Dual Link System/ HRP, Dako K4061, antibodies: Polyclonal Rabbit Anti-Human Calcitonin, Dako A0576, Polyclonal Rabbit Anti-Human Thyroglobulin, Dako A0251. All kits were sourced from Dako North America Inc., Carpinteria, CA, USA. Immunohistochemical staining followed the protocols of Wehner et al	From a few hours to 22 days after death	In comparison with the studies by Wehner et al.: earlier negativization of immunoreactions for insulin and glucagon	Immunohistochemical detection of different antigens promising in forensic practice
[27]	3 corpses. Information was collected relating to age, height, weight, date and cause of death, time elapsed and environmental conditions of conservation of the body (refrigerated or not) from death to arrival at the facility. The bodies were placed, 2 in a prone position and 1 supine, undressed, outside in a dry and shaded wooded area with a cool to temperate climate (38–77°F)	Skin collected from 4 sites: scalp, sole of the foot, hypostatic region of the trunk and non-hypostatic region of the trunk	Histology: hematoxylin/eosin stain No immunohistochemical investigation	Up to 7 days (samples collected each 12 h in the first 2 days, then every 24 h for the following 4 days)	Macroscopically, no significant differences were highlighted. The three major histological changes were: focal dermal-epidermal separation, necrosis of the eccrine duct (appearing between 4 and 7 days), and degeneration of the dermis (in non-truncal skin biopsies on day 2)	Although the study presents the limitation of the small number of subjects studied and the presence of uncontrolled extrinsic factors, the histological results from selected skin biopsies can be useful in estimating early PMI (necrosis of the eccrine glands and dermal degeneration)
[28]	500 corpses with known times of death and different environmental conditions and causes of death. Cases with pancreatic or cerebral pathologies diagnosed in life were excluded	Pancreas Brain	Anti-somatostatin and anti-glial fibrillar acid protein antibodies (monoclonal mouse anti-human GFAP)	From 1 to 23 days after death	Somatostatin: positive staining within 2 days after death; negative immunoreaction after 11 days GFAP: colourable in the frontal cortex within 3 days of death; constant negative immunoreaction after 14 days	The accuracy, practicability, and cost-effectiveness of immunohistochemical methods for delimiting the time of death make them likely candidates for routine forensic practice, especially when longer time since death is suspected
[29]	10 corpses	Gingival tissues	Types I and III anti-collagen antibodies: Type 1 anti-human collagen antibody (Millipore, Merck S.p.A., Milan, Italy) and with the anti-human collagen type III antibody (Sigma-Aldrich, St. Louis, Missouri, USA). Subsequent incubation with secondary antibody conjugated with peroxidase for 1 h at room T (Histofine immunohistochemical staining kit, Nichirei Biosciences INC, Tokyo, Japan). Signal detection with DAB following the manufacturer’s protocol (Histofine Immunohistochemical staining kit, Nichirei Biosciences INC, Tokyo, Japan)	Samples collected at: 1–3 days, 4–6 days, 7–9 days after death	Gradual degradation of the extracellular matrix in suboral connective tissue. PMI was related to increased nuclear chromatin condensation and cytoplasmic vacuolization in both epithelial and connective tissues	Potential for morphological characteristics (histological and ultrastructural) and immunohistochemical markers (type I and II collagen) of gingival tissues to estimate the time of death more accurately
[30]	10 corpses	Gingival tissues: maxillary gingiva adjacent to the first incisors	Immunohistochemical distribution and mRNA expression of hypoxia inducible factor (HIF-1a) Primary antibody against HIF-1a (Invitrogen, Thermo Fisher Scientific, Monza, Italy) Secondary anti-rabbit antibody, followed by diaminobenzidine tetrachloride (DAB)	Different time frames studied in an overall PMI ranging from 1 to 10 days: 1–3 days, 4–5 days, 8–9 days	Time-dependent correlation of the HIF-1° protein and its mRNA with the different time intervals from death: 1–3 days: High signal of the HIF-1° protein mainly localized in the basal layer of the oral mucosa 4–5 days: the signal gradually decreases 8–9 days: the signal is not detected	Interesting potential utility of techniques based on immunohistochemistry as important complementary tool to be used in forensic investigations
[31]	Rats: 40 adult male albino rats (weight 230–260 g), sacrificed after 1 week of acclimatization in a cage with 50 mg/kg pentobarbital Man: 40 bodies from court cases with known times of death (within the first 24 h). 23 males and 17 females ranging in age from 18 to 50 years	HMGB1 in serum and full thickness skin	Biochemical, histological and immunohistochemical study on blood and skin Histology with hematoxylin–eosin Immunohistochemistry with HMGB1 rabbit polyclonal antibody (Abcam, Cambridge, MA, USA; dilution 1:200) and secondary antibody conjugated with peroxidase	Rats: samples at 0.3, 12, 24 h Corpses: I: 0 h II: < / = 3 h III: 4–6 h IV: 7–12 h V: 13–24 h	Significant increase as a function of time in the serum levels of HMGB1, together with its overexpression in the skin. In parallel, histological changes in the epidermis, dermis and hypodermis were analysed	HMGB1 could be a post-mortem marker to provide a precise and feasible method for estimating PMI. However, further studies are needed to analyse different time intervals and other physical factors that can influence post-mortal levels of HMGB1 in different tissues

[32]	42 adult albino rats weighing 200–220 g (12 weeks of age) were sacrificed by spinal dissection	Heart Kidneys Testicles	Gene expression analysis of MDA, SOD, GSH, HMGB1; immunohistochemistry and histopathology of bcl2	Samples at: 0, 12, 24, 48, 72, 96 and 120 h after death	PMI is correlated with different tissue levels of MDA, SOD, GSH. HMGB1 exhibited increased post-mortem gene expression, peaking at 48 h post death. Obvious time-dependent histopathological changes were observed in all organs until 5 days after death. Oxidizing agents and antioxidants are related to PMI up to 120 h after death. BCL2 began to decline at 24 h and became negative 96 h after death	Expression of the HMGB1 gene can be used for estimating the PMI, as it shows time-dependent changes in the form of a progressive increase. Oxidizing agents and antioxidants and the immunohistochemical expression of BCL2 show variations that can be correlated with the passage of time since death
[33]	24 adult albino rats weighing 150.55 ± 8.56 g were sacrificed by cervical dislocation	Skin Muscles	Histology: hematoxylin / eosin stain No immunohistochemical investigation	8 groups: 0.8, 16, 24, 32. 40, 48 and 72 h after death	Significant differences were found in epidermal nuclear chromatin. The highest intensity was detected at 40 h The nuclei of the sebaceous glands and the condensation of nuclear chromatin showed dramatic decrease Striation of subcutaneous muscles can be identified between 8 and 16 h after death, while initial skeletal muscle degradation processes begin at 16 h	Histopathological changes in the subcutaneous muscles can be considered a tool for the determination of the PMI. The histopathological changes in the skin are similar to previous studies but appeared later than the others. This diversity can be attributed to different environmental factors This study proposes the use of morphometric parameters of the skin and underlying muscles to evaluate the time of death (early and late), while also considering various environmental factors and different tissues
[34]	31 corpses	Gingival tissues	Histology exams No immunohistochemical investigation	3 subgroups: 0–8 h (n 10); 8–16 h (n 10); 16–24 h (n 11)	The initial epithelial changes were homogenization and eosinophilia, with cytoplasmic vacuolation, epithelial crushing, ballooning, loss of nuclei and suprabasilar cleavage in the late interval (16–24 h). Nuclear changes such as vacuolisation, anorexia, pyknosis and karyolysis became increasingly evident with the lengthening of PMI. Homogenization of collagen and vacuolation of fibroblasts also appeared. The onset of decomposition at the cellular level appeared within 24 h of death, and other features of decomposition were observed thereafter	The histological changes that occur post-mortem in human gingival tissues seem to be a useful method for estimating the time of death in the first PMI (0–24 h). However, further studies are needed to verify, refine and expand these initial findings. Despite some constraints, such as the limited number of subjects and the relatively short time period of the post-mortem period, the present study demonstrated the potential of this method as a tool to be used in forensic practice Further research with larger samples and an extended time frame might help forensic professionals in the estimation of a precise hour of death
[35]	24 samples from patients undergoing extraction of third molars for orthodontic reasons	Dental pulp	Histology: hematoxylin/eosin stain and tricromica di masson No immunohistochemical investigation	The 24 samples were divided into 4 experimental groups: 1: group 1 ◊ 6 dental pulps 24 h after extraction 2: group 2 ◊ 6 dental pulps 3 months after extraction 3: group 3 ◊ 6 dental pulps 3 months after extraction 4: group 4 ◊ 6 dental pulps 6 months after extraction	Histological transformation of the pulp as the PMI increased Qualitative analysis: at 24 h, all structures were recognizable. With the increase in PMI, characteristic morphological changes were observed for each time interval. The quantitative analysis showed a significant decrease in nuclei as the PMI increased in contrast to the percentage of collagen fibres, which increased in relation to the PMI (significant differences, especially between 24 h and 6 months and between 3 and 6 months). No significant differences at 1 month	The study proposes the use of dental pulp in the estimating of PMI, considering a longer PMI than in previous studies, of up to a PMI of 6 months The qualitative and quantitative results support the use of this methodology in the PMI estimation to be applicable to forensic cases. Further research should focus on the use of a higher number of samples and in different environmental conditions to allow for a wider application of the methodology and possibly to help narrow down PMI time estimates
[20]	32 corpses	Dental pulp	Histology: hematoxylin/eosin stain No immunohistochemical investigation. Count of odontoblasts	Extraction of 1 tooth with only one root every 24 h from the jaw. Stored in an open plastic bag for 5 days Cases divided into 2 groups: 15 at room temperature (23 °C) and 17 at refrigerated temperature (4 °C)	The number of odontoblasts drops with the passage of time since death. The average drop in odontoblast density is 130 per square millimetre per hour at room temperature, and 120 per square millimetre per hour in refrigerated conditions. The pulp is free of odontoblasts 5 days after death The study also showed that lower temperatures do not significantly slow the breakdown of odontoblasts	The number of odontoblasts and their histological appearance may be an additional parameter in the estimation of the time of death. More cases are needed to provide other exploratory possibilities, for example, how odontoblasts degenerate and degrade as a function of time
[21]	74 corpses (adults aged 20 to 97: 40 men and 34 women) Biopsies: 22 patients aged 7 to 90 years of both sexes who donated bone marrow or who underwent biopsies	Bone marrow (BM)	TRAP detection as a marker of osteoclasts by formalin histochemistry	PMI from 0 to 5 days in cases of pathological anatomy and from 2 to 42 days in forensic autopsies	Osteoclasts remain TRAP-positive for 7 days post-mortem, albeit with markedly reduced staining intensity compared to biopsies Since TRAP-SI has not been correlated with the duration of PMI (individual variability of TRAP positivity in osteoclasts present in vivo), TRAP staining of osteoclasts in BM cannot serve as a tool to determine the time of death of a patient	TRAP staining is an appropriate tool for detecting medullary osteoclasts in autopsy specimens However, due to the individual variability of TRAP positivity in osteoclasts, staining alone cannot serve as a tool for determining the time since death
[22]	Man, 29 cadavers (17 males, 12 females) with an average age of 74 at death (range 54–94). In all cases, the death was due to natural causes	Glands of skin on the ventral surface of the limbs	Analyses: 1. Cytochemistry with hematoxylin and eosin 2. Immunohistochemistry (S-100, CEA, Cytokeratin, ASM) 3. Ultrastructural electron microscope	Withdrawals 3, 6, 9 and 12 h after death	Electron microscopy identified specific changes for each chronological phase: reduction of intracellular glycogen in light cells and secretory granules in dark cells are signs of the first stage after death (3 h); mitochondrial dilatation and rarefaction of the crypts in light and dark cells at the second stage (6 h); and rarefaction of microvilli in light and dark cells at the last stage (12 h) Cytochemistry and immunohistochemistry provide useful information not for the whole chronological stage considered, but for single phases: 3 h for hematoxylin–eosin and 6 h for alcian-PAS	Potential use of optical, electron and immunohistochemical microscopy techniques in the determination of post-fatal time intervals, using the morphology of the eccrine sweat glands. The study proposes the combined use of these methods on a larger number of samples to deepen their use in forensic practice
[36]	Rats, 23 male mice (25–30 g), sacrificed with an intraperitoneal overdose of ketamine and xylazine	Skeletal and cardiac muscle	Histological study with hematoxylin–eosin and immunohistochemical for the research of caspase 9 and caspase 3 ◊ monoclonal rabbit anti-caspase 9 (Abcam, code ab222231) and monoclonal rabbit anti-caspase 3 (Abcam, code ab224271)	0, 4, 8, 12, 24 and 72 h after death 6 groups: -N. 4 a 0 h -N. 4 a 4 h -N. 4 a 8 h -N. 3 a 12 h -N 4 a 24 h -N 4 a 72 h	Skeletal muscle: Increase in the immunoreactivity of caspase 9 from 4 to 8 h, followed by stable levels, even if slightly decreased, for up to 24 h; significant reduction at 72 h. Immunoreaction for caspase 3 absent up to 4 h, the highest level at 8 and 12 h, and progressive decrease at 24–72 h The decrease of both at 24 and 72 h is in line with the appearance of increasing alterations in tissue morphology at hematoxylin and eosin. The apoptotic pathway was also confirmed: following initiator caspase 9 activation and then executing caspase 3 activation (statistically significant 4 h after death) Skeletal muscle matching matrix proteins degrade more consistently as PMI progresses than heart muscle	Usefulness of the combined immunohistochemical analysis of both caspases in the progression of time from death, in estimating the PMI
[26]	12 healthy horses	Brain, skeletal muscle, liver	Histology: hematoxylin/eosin stain No immunohistochemical investigation	0, 1, 2, 4, 6, 12, 24, 36, 48, 60 and 72 h after death The samples were stored at 22 °C and 8 °C	From 5 to 7 parameters associated with autolysis were identified for each tissue and quantified based on the percentage of the field involved under the microscope The changes were most noticeable in the liver and muscle tissues in the first 72 h post-mortem. The most evident characters at both temperatures in the first 72 h are: individualization of hepatocytes and separation of the bile duct epithelium from the basement membrane. In the interruption of the continuity of myofibers, hypereosinophilia and loss of striation The brain changes were highly variable	High statistical correlation between autolysis and the time/temperature variables, can be useful for establishing histological algorithms for identifying the PMI
[37]	30 corpses: 24 males and 6 females with ages between 15 and 64, died from various causes	Skin of the anterior thoracic region (epidermis and dermis with annexes)	Histology: hematoxylin/eosin stain No immunohistochemical investigation	3, 6, 9, 12, 15, 18, 21 and 27 h after death	The epidermis and dermis appeared normal for 6 h after death, and after this period the degenerative changes began 6 to 9 h after death, degeneration began in the dermis, and by the end of 18 h, the dermis began to disintegrate. The sweat glands appeared normal for about 3 to 4 h. For 18 h after death, the sebaceous glands and hair follicles appeared normal. After this period degeneration began	The histological changes found in the present study are similar to those described in previous literature. However, the changes seem to appear earlier due to environmental differences, which, as is well known, affect the decomposition. The analysis of the morphology and degeneration times of the sebaceous glands and hair follicles appeared useful in this PMI study. The parameters described can therefore represent a useful method for helping to estimate the time of death in the first post-mortem period
[31, 38]	8 cadavers aged between 22 and 33 years, including 2 women and 6 men, BMI 21.2–23.3	Full thickness skin with subcutis at sternum level	Histology: hematoxylin/eosin stain No immunohistochemical investigation	Autopsy performed in all cases within 4 h of death. A 10 cm × 2 cm piece of skin was taken from each corpse and refrigerated at 4–6 °C. Samples were taken from the piece at different PMIs: 4 h, 6 h, 12 h, 18 h, 24 h, 36 h, 48 h, 60 h, 72 h, 84 h, 96 h, 6 d, 8 d, 10 d, 12 d, 16 d, 20 d, 24 d, 28 d and 32 d	The epithelial cell nucleus condensed within 24 h of death and cell lysis was exhausted after 20 days The changes in the dermis occurred later than those in the epidermis (72 h), but after the onset of the epidermal changes, the processes in the dermis were more rapid. At 16 d, the layers had homogenized. The epidermis and dermis had completely separated 24 days after death. The changes in the sweat glands appeared earlier (24 h) and disappeared later (32 days); the sebaceous glands and hair follicles began to undergo degenerative changes at 96 h after death, and at about 20 d, only their outline remained	Post-mortal histological changes in the skin occur at specific times. Therefore, they could be used to help deduce the time of death Comprehensive observation of changes in the composition/structure of the skin is important to fully analyse the possible times of death

In this study, we investigated the possibility of estimating PMI using changes in the immunohistochemical expression of the HMGB1 protein and its related factors, Beclin1 and RAGE. HMGB1 is a nuclear, non-histone DNA-binding protein, characterized by high electrophoretic mobility on polyacrylamide gels. In mammals, there are four distinct HMGBs (HMGB1-4). However, HMGB1 is by far the most prevalent and ubiquitously expressed. It is a highly mobile protein found inside the nucleus of cells, where it modulates chromatin structure and increases the accessibility of binding sites to regulatory elements, such as transcription factors and nucleosomes [31–34].

HMGB1 comprises 214 amino acid residues, with a sequence identity of 99% in mammals. The molecule is divided into two positively charged DNA-binding domains, known as HMG-box A and box B, as well as a strongly negatively charged C-terminal tail, containing 30 glutamic and aspartic acid residues that are repeated 30 times. Lysine residues account for 20% of the amino acids in the whole molecule. Because of the sequential order and composition of a high number of negatively and positively charged amino acids, HMGB1 is a unique bipolarly charged molecule, a feature that earned it the additional designation “amphoterin” [31–35].

When HMGB1 is passively released after cell death or actively secreted into the extracellular space, it becomes a strong mediator of inflammation. Nuclear HMGB1 translocates from the nucleus to the cytoplasm in response to cell activation or injury, where it participates in inflammasome activation and pyroptosis, promoting autophagy by binding to Beclin1 and inhibiting apoptosis. Although numerous aspects of these key intracellular activities have been clarified, there is still more to learn about HMGB1 biology, which is vital for both cell survival and cell death.

## Conclusions

The only current study in the literature that deals with analysing the trend of HMGB1 at intracellular levels is that by El-Din et al. [15]. Although these authors observed a decrease in the cytoplasmic levels of the HMGB1 marker as time passed after death, they did not characterize its translocation from the nucleus to the cytoplasm. However, in our investigation, this translocation was highlighted and seemed to occur around 12 h after death. Thus, the overexpression of nuclear HMGB1 indicates that death occurred within the last 12 h, the negativization of the marker at the nuclear level with a high intensity of the marker at the cytoplasmic level indicates that death occurred between 12 and 36 h previously, while the negativization of the marker at the cytoplasmic level suggests that more than 48 h have elapsed since death.

Beclin1 and RAGE do not appear to have been investigated previously as potential PMI indicators. In this study, we found that the cytoplasmic levels of these proteins (in keratinocytes and annexes) decreased over time. The negativization of RAGE and Beclin1 may imply that more than 24 and 36 h have passed since the time of death, respectively. There is a scarcity of evidence in the literature on post-mortem skin changes relating to time since death. The current study aimed to perform a semi-quantitative analysis of three separate intracellular protein markers: HMGB1, RAGE and Beclin1. Based on the findings of this investigation, these may all be useful markers for estimating PMI using an immunohistochemical approach. Consequently, it may be inferred that these results have laid the groundwork for supporting immunohistochemistry research in the context of thanatochronology. The application of the procedure to a larger case series, taking into account additional PMIs, as well as the implementation of a similar study on human tissues, would be beneficial in generating reliable and practical protocols to be used in a forensic routine.

## Key points

1. Immunohistochemistry can be used as a method for determining the PMI
2. Post-mortem skin samples of 20 adult mice were analysed using immunohistochemistry
3. Skin HMGB1 proteins and associated components could help with PMI determination

4. Different expression patterns of the studied proteins correspond to different PMIs
